# Suicide prevention for youth - a mental health awareness program: lessons learned from the Saving and Empowering Young Lives in Europe (SEYLE) intervention study

**DOI:** 10.1186/1471-2458-12-776

**Published:** 2012-09-12

**Authors:** Camilla Wasserman, Christina W Hoven, Danuta Wasserman, Vladimir Carli, Marco Sarchiapone, Susana Al-Halabí, Alan Apter, Judit Balazs, Julio Bobes, Doina Cosman, Luca Farkas, Dana Feldman, Gloria Fischer, Nadja Graber, Christian Haring, Dana Cristina Herta, Miriam Iosue, Jean-Pierre Kahn, Helen Keeley, Katja Klug, Jacklyn McCarthy, Alexandra Tubiana-Potiez, Airi Varnik, Peeter Varnik, Janina Žiberna, Vita Poštuvan

**Affiliations:** 1 Department of Child and Adolescent Psychiatry, Columbia University-New York State Psychiatric Institute, New York, NY, USA; 2 Department of Health Sciences, University of Molise, Campobasso, Italy; 3 Department of Epidemiology, Mailman School of Public Health, Columbia University, New York, NY, USA; 4 National Centre for Suicide Research and Prevention of Mental III-Health (NASP), Karolinska Institute, Stockholm, Sweden; 5 WHO Collaborating Center for Research, Methods Development and Training in Suicide Prevention, Stockholm, Sweden; 6 Department of Psychiatry, School of Medicine, University of Oviedo; Centro de Investigación Biomédica en Red de Salud Mental, CIBERSAM, Oviedo, Spain; 7 Feinberg Child Study Centre, Schneider Children’s Medical Centre, Tel Aviv University, Tel Aviv, Israel; 8 Vadaskert Child and Adolescent Psychiatric Hospital, Budapest, Hungary; 9 Department of Developmental and Clinical Child Psychology, Institute of Psychology, Eotvos Lorand University, Budapest, Hungary; 10 Department of Clinical Psychology, Iuliu Hatieganu University of Medicine and Pharmacy, Cluj-Napoca, Romania; 11 Section for Disorders of Personality Development, Clinic of Child and Adolescent Psychiatry, Centre of Psychosocial Medicine, University of Heidelberg, Heidelberg, Germany; 12 Research Division for Mental Health, University for Medical Information Technology (UMIT), Hall, Tyrol, Austria; 13 Department of Psychiatry and Clinical Psychology, University Medical Centre, Université de Lorraine, Nancy, France; 14 National Suicide Research Foundation, Cork, Ireland; 15 Estonian-Swedish Mental Health & Suicidology Institute, Centre for Behavioural Sciences, Tallinn University, Tallinn, Estonia; 16 Department of Population Studies, Tallinn University, Tallinn, Estonia; 17 Slovene Center for Suicide Research, Andrej Marušič Institute, University of Primorska, Koper, Slovenia

**Keywords:** Youth, Adolescents, Mental health, School-based, Awareness program, Suicide prevention, SEYLE, Intervention

## Abstract

**Background:**

The Awareness program was designed as a part of the EU-funded Saving and Empowering Young Lives in Europe (SEYLE) intervention study to promote mental health of adolescents in 11 European countries by helping them to develop problem-solving skills and encouraging them to self-recognize the need for help as well as how to help peers in need.

**Methods:**

For this descriptive study all coordinators of the SEYLE Awareness program answered an open-ended evaluation questionnaire at the end of the project implementation. Their answers were synthesized and analyzed and are presented here.

**Results:**

The results show that the program cultivated peer understanding and support. Adolescents not only learned about mental health by participating in the Awareness program, but the majority of them also greatly enjoyed the experience.

**Conclusions:**

Recommendations for enhancing the successes of mental health awareness programs are presented. Help and cooperation from schools, teachers, local politicians and other stakeholders will lead to more efficacious future programs.

## Background

### Suicide prevention in youth

Every completed suicide has a devastating effect, but when a young life is cut short, the shock is oftentimes even greater. Suicide is a complex phenomenon, thus, the prevention of it needs to be tailored accordingly [1,2]. Prevention can occur on both the individual and societal level, with the most effective strategies being a combination of efforts [1,3]. An obstacle in the effort to combat suicide is the difficulty in identifying exactly which at-risk individuals will commit suicide [4-6]. Consequently, by informing the public and encouraging a general awareness of mental health problems including suicide, an increased alertness and responsiveness to suicidal individuals will follow [7]. In an effort to make such suicide preventive strategies effective and culturally appropriate, it is important to consider local attitudes toward suicide, and how to target suicide prevention and mental health interventions. Furthermore, it is imperative to take into account the feelings of pain and grief experienced by any community or individual that has encountered a suicide.

Stigma, developmental changes and peer pressure lead to adolescents being particularly in need of specifically tailored preventive strategies [8,9]. Youth rarely look for help from professionals. The reasons for this are many and difficult to disentangle; perhaps the healthcare system is not adequate, or there are none or few mental health professionals available, but it can also be related to developmental changes, increasing sense of self-autonomy and attitudes toward adult intervention. Young people may not ask for help because they see it as a failure in the process of becoming self-sufficient [10-12]. They may believe growing up means being able to cope with their problems by themselves. Perhaps they consider their problems unique and therefore unsolvable, be it by professionals or anyone else. Oftentimes young people are reluctant to look for professional help because of the stigma of mental illness and, for similar reasons, they may also be afraid to address the issues of mental pain to their peers [13]. Thus, it is important to consider all of these factors when creating suicide prevention programs for youth.

### Awareness programs for youth

It is well known that the majority of young people will not actively seek help from professionals, parents, teachers, and oftentimes not even from their peers [9]. With this in mind, how can youth suicide effectively be prevented? In 2002, the World Psychiatric Association (WPA) launched a 9-country pilot study in order to raise the knowledge and awareness about mental health in young people. The assumption was that sound information would facilitate communication about mental health concerns, without raising unrealistic expectations about professional help that was generally unavailable [14,15]. In the WPA 9-country study, the awareness campaigns were locally designed and, thus, culturally adjusted to be acceptable for the local population. The results showed that it was possible to change attitudes, including those about suicide, by influencing the behavioral responses of the pupils and parents that partook in the study with slightly poorer results for the participating teachers. Building on that pilot study, an awareness program for adolescents was designed for the Saving and Empowering Young Lives in Europe (SEYLE) study, funded by the European Union within the 7^Th^ Framework Health Theme.

### The SEYLE study

SEYLE is a randomized-controlled intervention trial (RCT) designed to assess the effects of three different health-promoting intervention programs in comparison with a control group in which a minimal intervention was carried out. The study methodology has been described previously in detail [16]. The intervention programs consisted of:

1. Awareness Program – a health promotion program, designed to empower pupils by increasing their awareness of mental health, as well as healthy/unhealthy behavior and teaching them skills to diminish unhealthy behaviors [developed for the SEYLE study by Columbia University and Karolinska Institutet/National Centre for Suicide Research and Prevention of Mental Ill-Health (NASP)].

2. QPR (Question, Persuade & Refer) – a gate keeping program designed to educate teachers and other school-based adults in identifying at–risk adolescents and referring them to mental health facilities [17].

3. ProfScreen – screening by professionals for the purpose of identifying pupils at high risk for mental illness and/or suicidal behavior. The program includes a referral procedure, wherein pupils identified as at-risk of mental illness or suicidality were referred to mental health treatment; this program was specifically tailored for the SEYLE study, by the Heidelberg University and Karolinska Institutet/NASP research groups.

4. Minimal Intervention (control group) – providing pupils with information materials (posters on the classroom walls), containing basic information about mental health (e.g., warning signs of crisis and mental illness, how and where to seek help). This intervention served as the control arm.

In the SEYLE study, effectiveness of the respective interventions on adolescents was compared between the interventions and the control group.

#### Awareness program in the SEYLE study

In the SEYLE study, the Awareness program was developed to target adolescents between 14–16 years old and to meet their mental health-related needs. The strategy of the program was to integrate different types of learning in order to guide the adolescents through difficult topics. One of the most effective ways to target changes in youth is to combine both a cognitive and emotional training program [18,19]. Cognitive learning was achieved through lectures about mental health and mental disorders, and experiential and emotional learning through role-play sessions, as well as an overall hands-on approach to sensitive issues. The four-week interactive program prescribed a stimulating environment without involvement of the regular schoolteachers/staff in order to diminish concerns of being judged. Guided by a trained instructor and at least one assistant, the adolescents were given an opportunity to learn from peers, whilst reflecting on personal experiences and problem-solving techniques by actively using their newly acquired skills in the role-play sessions [20,21].

Before the implementation of the program and during the preparatory phase, site-visits were made by members of the SEYLE consortium steering group in order to ascertain that the protocol was followed. The site leaders, along with the coordinators of the Awareness program and the instructors appointed to lead the role-play sessions were trained in the many facets of the study methodology; the procedures were stipulated in a detailed 31-page instruction manual [21]. The Awareness coordinators were child psychiatrists or psychologists, many of whom had prior experience with psychodrama or role-play.

**Figure 1:**
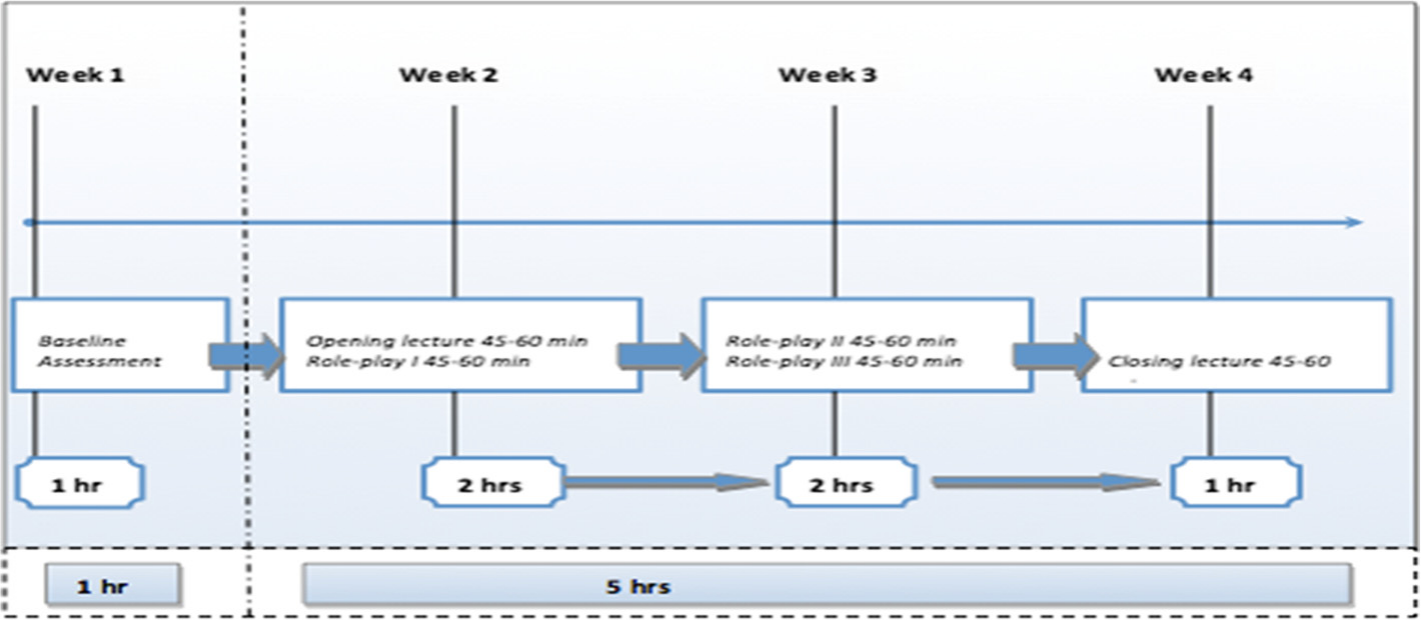
Awareness program timeline in the SEYLE study.

The program started with a baseline assessment. The core of the program consists of an opening lecture, three role-play sessions, and a closing lecture with a discussion. In the SEYLE study, each session lasted 45–60 min and the whole program was carried out during four weeks, in a total of five hours plus one additional hour for the baseline questionnaire that served as an introduction and first contact with the students (Figure 1).

**Figure 2:**
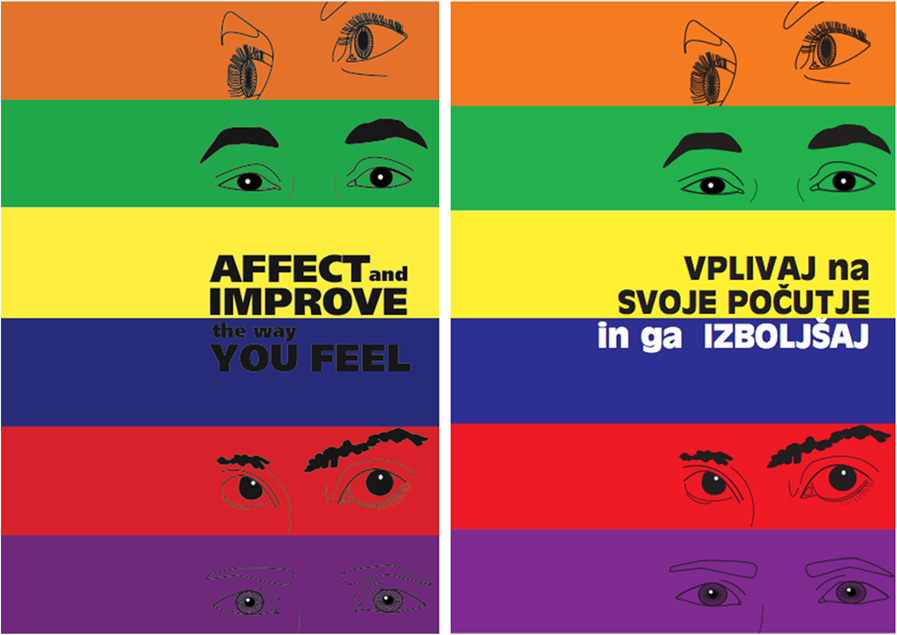
Booklet cover pages (English and Slovene booklets).

A didactic and pedagogical booklet (Figure 2): “Affect and Improve the Way You Feel” [20], specifically created for the Awareness program was distributed to all students. The booklet contained the following themes:

1) Awareness of mental health

2) Self-help advice

3) Stress and Crisis

4) Depression and Suicidal thoughts

5) Helping a troubled friend and

6) Getting advice: Who to contact

The booklet of approximately 25 pages was designed for the SEYLE study in close collaboration with a graphic designer who had prior experience in public mental health research and prevention. It was translated, back translated and, when needed, culturally adapted to fit the local languages of the participating sites. In Israel the booklet was translated into both Hebrew and Arabic. The booklet was designed so that it could be kept as a future resource for the pupils at the end of the Awareness program. The content of the booklet served as a framework for the role-play sessions and was introduced to the pupils during the opening lecture in a power point presentation. Similar information was also briefly summarized in the six posters that were hung in the classrooms.

In the SEYLE study it was recommended that 10–15 students per instructor participate in the role-play sessions. Through role-play sessions and the ensuing discussions, the students learn about mental health related problems, whilst developing a set of problem-solving skills to assist them in distress, as well as the ability to identify circumstances in which the skills should be applied. They get the opportunity to identify reasons for, and ways to prevent the escalation of problems and to explore the potential effects on the people directly and indirectly involved. In order for role-play to be an effective tool, all questions and thoughts expressed by the pupils need to be thoroughly discussed. This provides the pupils with an opportunity to explore specific situations (e.g. being bullied in school, a family crisis, moving to a new town, feelings of depression and suicidal thoughts) that could otherwise appear threatening or difficult in an unsafe environment. They were taught and given the chance to practice how to express empathy, to appreciate other peoples’ perspectives and how to stand up against peer-pressure. The sessions gave also the opportunity to talk about the responsibility of school staff and adults, for example in the case of bullying. Finally, in the closing lecture, all the topics discussed are summarized by using the same power point presentation as in the opening lecture. In this final meeting with the students, particular attention is given to the contact information found at the end of the booklets. In every SEYLE site, local information with the names and telephone numbers of people in the healthcare system and other community-based support networks were provided for students to seek help.

### The instructor’s role

In addition to the Awareness coordinators, the program was carried out by a team of competent instructors (also called facilitators). The procedures manual stated that the instructor should hold a Masters or higher degree in psychology, public health, social work, pedagogy, or of an equivalent discipline. It was also recommended that the instructor have at least one assistant during the labor-intensive role-play sessions. Some sites even decided to hire professional psychodrama therapists to lead the role-play sessions. The instructors were asked to keep a journal during the time of the intervention program, keeping track of any and all deviations from the protocol and all cultural adaptations.

#### Aim

In this descriptive study, the Awareness program field experiences are captured by using first-hand information from the 11 SEYLE sites, as such, generating recommendations and enhancing the future potential of such a suicide prevention strategy.

#### Ethical permission

Ethical permission for the project, including the permission to follow up individual pupils, was obtained in each one of the eleven participating countries by their respective Research Ethics Committees, namely: Austria: Ethikkomission der Medizinischen Universität Innsbruck; Estonia: Tallinna Meditsiiniuuringute Eetikakomitee; France: Comité de Protection des Personnes Sud-Méditerranée II; Germany: Ethikkommission Medizinische Fakultät Heidelberg; Hungary: Egészségügyi Tudományos Tanács Titkárság, Tudományos És Kutatásetikai Bizottság; Ireland: Clinical Research Ethics Committee of the Cork Teaching Hospital; Israel: Helsinki Committee at the Rabin Medical Center; Italy: Comitato Bioetico Di Ateneo, Università Degli Studi Del Molise; Romania: Comisia De Etica, A Universitatii De Medicina Si Farmacie, Cluj Napoca; Slovenia: Komisija Republike Slovenije za medicinsko etiko; Spain: Comité Ètico de Investigación Clinica, regional del Principado de Asturias.

## Methods

### Sample

The SEYLE Awareness program was carried out within well-defined catchment areas in eleven countries: Austria, Estonia, France, Germany, Hungary, Ireland, Israel, Italy, Romania, Slovenia and Spain. In those eleven catchment areas, 179 schools were randomized into one of the four non-overlapping intervention study Arms. The participation rate of pupils was 88% at baseline. A total of 12, 395 pupils from both metropolitan and micropolitan areas participated in the study, of which 6799 were females and 5529 were males (67 subjects had missing gender data), with a mean age of 14.9 ± 0.9. Description of the methodology and material employed is given in another paper [22]. A total of 3016 pupils participated in the Awareness program Arm (55.2% females and 44.8% males).

In this paper, we examine the experiences and opinions about the Awareness program of the 11 SEYLE Awareness coordinators.

### Procedure

In order to examine the strengths and weaknesses of the program in this descriptive study, we asked all the Awareness coordinators the following set of open-ended questions about the program implementation.

1) What did you like most about the Awareness program?

2) What did you like least about the Awareness program?

3) What did the pupils like most about the Awareness program?

4) What did the pupils like least about the Awareness program?

5) How did the schools and teachers like the Awareness program?

6) What would you change in the Awareness program if you could?

7) What parts of the intervention needed to be culturally adapted for your specific country?

8) Was there a difference between the participating schools? Classes? In their willingness to participate, how they participated, what they thought, etc.

9) How much effort did the organisation of the Awareness program take?

10) In your opinion, was the effort worth the outcome?

Upon completion of the Awareness program, the coordinators in all sites were asked to answer the above-mentioned questions in writing.

#### Data analysis

The written answers to the open-ended questions were analyzed by the first and the last author of this paper independently, with the processing of the material performed in a number of steps. As recommended by Pope et al. [23] the coding process was conducted with researchers from different backgrounds (in psychology, public health and anthropology).

To begin, each response was reviewed independently by two assessors (VP and CW). Secondly, in order to identify emergent topics and to ascertain meaningful and broader themes, words and sentences were grouped together [23-25]. After distinguishing the themes, the two assessors independently scrutinised the whole material again before comparing their results. The interpretations were mostly congruent, but in the case of discrepancy regarding which theme an answer belonged to or having different opinions about the naming of the theme clusters, a third independent assessor (DW) was consulted and the final classification and grouping of responses into theme clusters was obtained with consensus.

Importantly, the themes describe multifaceted phenomena that are broad in nature, but for the purpose of analysis are grouped together [24]. Several themes describing similar topics were combined, e.g. *role play and expressing feelings* includes what the coordinators described as the possibility of practicing the expression of emotions through acting and *improved coordination (with schools and staff)* represents organisational difficulties such as scheduling with schools, meeting teachers and headmasters as well as recruiting staff. As a last step, a general description of the responses was written, serving as the basis for the results reported in this article. Issues that were voiced by some sites in particular are emphasised in the result description by adding the name of the country in parenthesis. The Awareness coordinators as well as the principal investigators of each of the sites were given the opportunity to comment on the interpretation of the responses.

## Results

### Strengths of the awareness program

**Figure 3:**
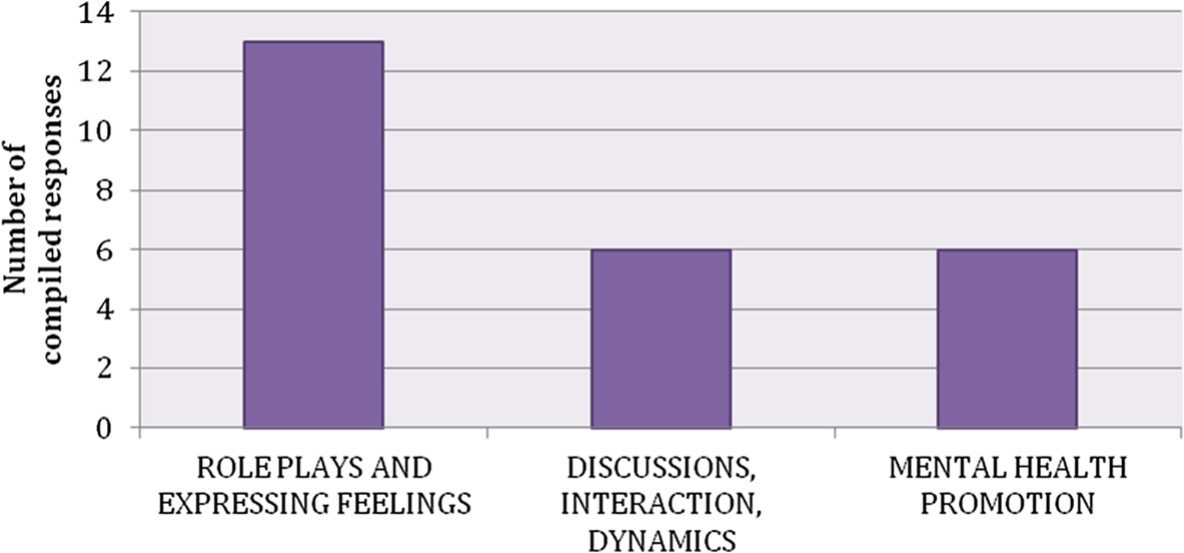
**Reasons for appreciation of the awareness program. **Due to the fact that the Awareness coordinators responses were compiled into theme clusters the charts show the number of compiled responses and not the percentage of responses.

The Awareness coordinators from all sites drew attention to the particular design of the program that gave space for discussing important mental-health related topics, which otherwise go unaddressed. In 6 of the 11 participating sites (Austria, Estonia, Germany, Hungary, Italy, Slovenia), the coordinators underscore that talking about mental health problems and emotions is still uncommon, shameful and stigmatised. Figure 3 shows the aspects of the Awareness program that were most appreciated across sites.

From the evaluation results, and as intended, the adolescents used the role-play as an opportunity to discuss their feelings, and they were eager for this kind of experience (Austria). Adolescents particularly appreciated the opportunity to talk about topics such as problem-solving, depression, anxiety, bullying (Austria, Germany, Israel), stress and crisis situations (Spain), pregnancy, conflicts with parents and teachers (Romania), and also suicidal behavior (Slovenia, Israel). The experience in France also showed that it was important that positive aspects of health were addressed. In Estonia, it was noticed that, in schools with a higher proportion of children with social problems, more serious topics emerged during the role-play sessions.

According to the Awareness coordinators, the program successfully promoted social networks in all countries. Pupils often reported that, when they are in distress, they do not have anyone to talk to (Hungary, Israel). The Awareness program addresses this problem in two ways: First, pupils are informed about different kinds of professional support. Second, the Awareness program promotes peer support. The importance of peer support is emphasised directly with guidelines on how to help a friend in need and indirectly by developing empathy. By participating in the program, the youth got to know each other in a deeper way, often realising that they were not alone with their problems, and that classmates, who they often didn’t know very well, shared similar problems (Hungary). Students also learned the importance of offering support to peers, instead of avoiding their problems, and learned how to do it more effectively. All countries report positive outcomes in this regard. In some cases, the Awareness program also contributed to stronger class bonds (Hungary) or an improved general school-climate (Israel) as reported by students to the instructors.

#### Interactive workshops as a means of prevention

The adolescents and instructors alike appreciated the interactive approach of the Awareness program. The relaxed atmosphere of the role-play sessions proved to be a good point of departure for discussion, and a way to approach the youths’ thoughts and feelings. The instructors often noticed that pupils had difficulties expressing their feelings in words (Austria, Germany, Israel, and Slovenia). The role-play sessions provided them with the opportunity to communicate, express and verbalise their feelings, not only to the facilitator, but also to their peers. They were able to overcome their fears of expression, and open up in a more relaxed way (Austria, Italy). The interactive approach engaged pupils, and they preferred it to the standard classroom set-up, or the ex-cathedra approach, which is still the predominant way of teaching in many schools across Europe. Not only the pupils, but also the instructors, liked the variety of verbal and written materials used in the Awareness program, as well as the more interactive components in contrast to the lectures (Austria). There were reports from all participating countries about students approaching the facilitators after the end of the program, telling them about their problems. Moreover, school counsellors noted that the Awareness program lead to the development of networks with the clinical sector, specifically by providing information on the treatment of pupils in distress, much to the benefit of the perceived quality of care in the schools (Slovenia).

The instructor from the Irish site gave the example of how a young boy actively used the booklet as a means to speak to his mother about his feelings and worries. The mother came to the school after one of the sessions to speak to the instructor; she had noticed a marked change in his mood and was very thankful. The instructor also noticed that the boy had become more vocal as the Awareness sessions proceeded.

Moreover, the instructors reported changes in the adolescents’ behaviors as the 4-week program progressed; it was evident that, from participating in the role-play sessions, the youth developed problem-solving skills when faced with different situations (Ireland). Additional analyses are required to learn how this potentially translates into everyday life and in preventing mental health problems.

The schoolteachers expressed the importance of having a person from outside the school-system to perform the program (Austria), avoiding possible distrust of more familiar instructors. The emotion that they could express their views and emotions in a safe environment, without prejudice and fear of ridicule, was a very powerful aspect (Ireland, Romania). Pupils indicated that they liked that the instructors were open-minded and young, and someone to whom the pupils easily could make a connection with and feel close to. With all of this in mind, it is very important to assemble, train and manage a team able to deliver this kind of program to young people (Ireland, Romania).

### The shortcomings of the awareness program and proposals for future modifications

The shortcomings of the Awareness program, as voiced by the instructors, mostly concern the lack of flexibility due to the RCT design and the tight time frame in the implementation of the workshops. It was difficult to assure that the needs of all pupils were met, or that all topics were equally addressed, explained and/or understood with the same depth. Some topics (e.g., serious mental health problems like depression or suicide) were more difficult to comprehend for some adolescents and, thus, a challenge for the instructors to convey in such a brief period of time.

The question of allocating more time for role-play sessions, e.g. 2 h instead of 45–60 min, was raised. The current program included an opening lecture that was considered by some to be too theoretical in nature and, consequently, not as well accepted as the interactive role-play sessions (Austria, Israel). Moreover, pupils thought the time frame for each session was too short (France).

#### Burden of the program for the school system

A potential obstacle to successfully implementing the Awareness program is the response of the school authorities, school staff, and their parents. In some cases, teachers and school staff were sceptical about the pupils’ motivation to participate in such a program. Ensuring that the entire teaching body appreciates the benefits and efforts of such a program is beneficial to the implementation (Ireland). As the Awareness coordinator typically was in touch with the school principal and guidance counsellors across their site, many coordinators underscored that it proved to be helpful when the principal and/or guidance counsellor were asked to inform all teaching staff about the program. It also happened that some parents and teachers refused or discouraged pupil participation in the program, because they would miss too many classes (Austria, France, Germany, Hungary, Israel, and Slovenia). It would be helpful therefore to place hand-outs with information about the Awareness program in the staff room in order to ensure familiarity among the entire teaching staff (Ireland). In fact, the benefits of this kind of intervention program may not be obvious to everyone, especially parents (Hungary, Slovenia). In some schools, the Awareness sessions were scheduled after school and since many pupils attend other after-school activities, this could have influenced participation rates in the program.

**Figure 4:**
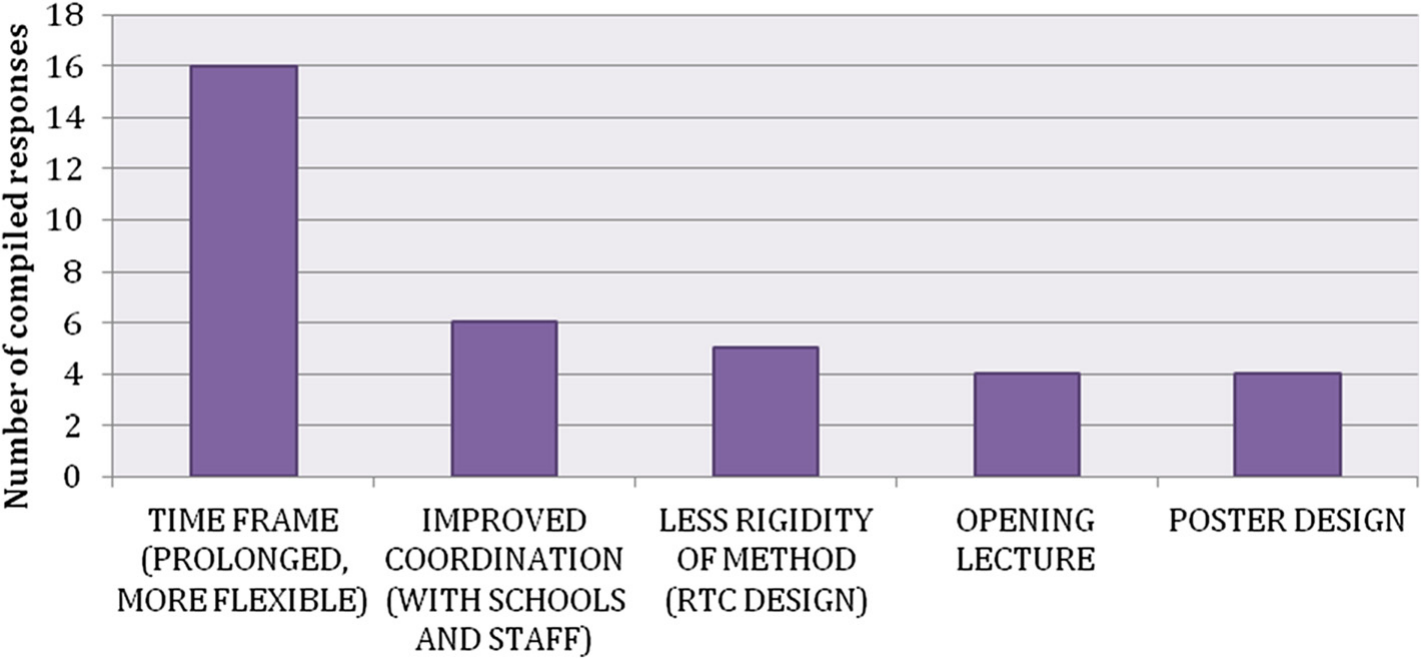
**Proposed modifications of the awareness program. **Due to the fact that the Awareness coordinators responses were compiled into theme clusters the charts show the number of compiled responses and not the percentage of responses.

#### A more holistic program and a longer time frame

The most important aspects that the coordinators wish to change in the program are shown in Figure 4 below. Many of them mention the short time frame of the program and the value of a more flexible schedule, as well as the advantage of a less rigid approach to the dissemination of content, expressing some reservation regarding the structure of the opening lecture and the somewhat intrusive posters. Instructors and students alike expressed the desire for an Awareness program that would last longer (Germany, Hungary, Ireland, Italy, Slovenia, and Spain), and for the structure of the program to be changed to two longer workshops, instead of three shorter ones (Estonia). Additionally, a wish to address other topics was expressed by the coordinators, such as: sexual behavior (Slovenia), sexual orientation (France), influence of emotions and thoughts on behavior (Romania), practice with behavioral techniques about how to talk to peers in distress (Hungary).

#### Materials and tools of interaction

In addition to the role-play, discussions and problem solving that were part of the SEYLE Awareness program, adding other kinds of interactive teaching could further strengthen the program. Among these learning from videos (Italy) expressive arts techniques or even action teaching were mentioned (Slovenia).

Moreover, in some countries (Germany, Slovenia) pupils did not like the posters, as their design or style was not appealing and was sometimes considered too intrusive. This issue can be dealt with by minimising the amount of text on them and by giving more attention to the design. One problem with the posters, in addition to their somewhat simple design, was that they were printed locally, and the quality of the prints varied greatly from site to site. The Awareness and instruction booklets were all printed in Sweden at a printing company and, consequently, were of high quality and uniform across sites.

### A cross-country comparable awareness program

In the SEYLE study, the Awareness program was implemented in an identical fashion across the 11 countries. According to the SEYLE protocol, the sites were encouraged to, if needed, culturally adjust the content of the role-play examples and to account for these adjustments by keeping a journal at the time of program. In some cases, some of the role-play examples from the instruction manual were not used or, if used, applied in a modified fashion. Nonetheless, all participating sites addressed the same topics, as it is important in a cross-country primary preventive RCT to have well-structured tools and clear guidelines on how to work with pupils, so that the instructors could be easily trained, and the obtained intervention results to be comparable across schools and countries.

#### Flexibility vs. uniqueness

The short time frame of the program (four weeks) was stipulated because of the SEYLE study research-design, aiming to compare different intervention outcomes. For future implementation of this kind of preventive program, the structure and the time frame of the intervention can also be tested, since it is often difficult to offer it in an identical way in different countries and school-settings. The Awareness program stimulates the pupils’ thoughts and feelings, as such, creating a need for a space for continuous discussion, something that should probably be integrated into the ordinary school curriculum. In the Hungarian case, a group of pupils decided to continue meeting together weekly to discuss their problems among themselves at the end of the program. Moreover, coordinators from most of the sites underscored that the program was more successful in those schools where the number of pupils per session was fewer, as well as when more time was given for discussion. It was interesting to note that, in Austria, girls were more interested and open than the participating boys and, in Ireland, boys offered better advice when taking part in the role-play sessions compared to girls, especially, around the topic of pregnancy. In Romania, pupils from smaller towns were more involved and had a lot of questions during the introductory lecture, whilst pupils from bigger towns had higher expectations, expressing views about mental health that they had read about on the Internet and in other sources. Schools with pupils of lower Social Economic Status (SES) had lower participation rates (Hungary) and some adjustments of the program had to be made according the type of school (Slovenia).

**Figure 5:**
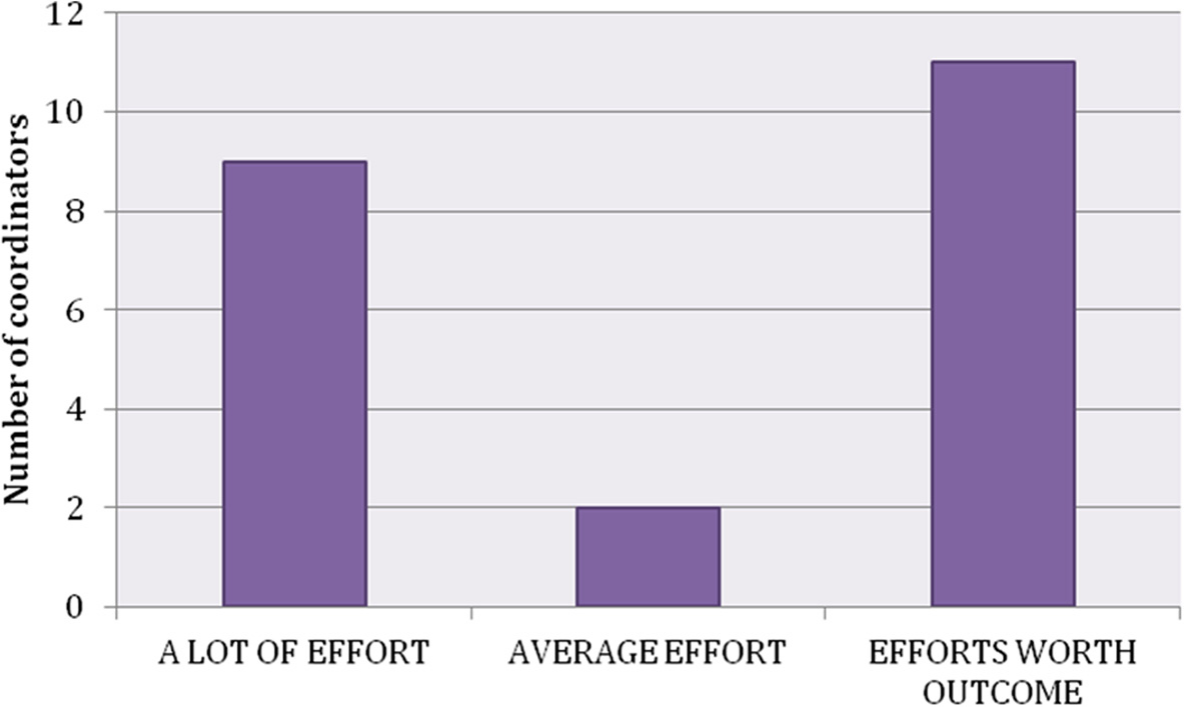
Organisational efforts and general satisfaction.

In summary, the key lesson is to uphold flexibility in discussion with the adolescents, taking into consideration the specific context of every classroom. Despite many challenges with the scheduling of the workshops in different schools and classes and other organisational efforts, all site Awareness coordinators reported that these were well worth it in relation to the satisfaction and appreciation expressed by the pupils (see Figure 5).

To overcome logistical difficulties as well as those related to the attitudes of the schools and parents, it is important that stakeholders, politicians, school-principals, teachers and parents understand the importance of such a mental health and Awareness intervention program, including its’ aims and design and that they support it (Israel, Germany, Hungary). It is also important to have close collaboration with other systems (e.g. social and health care system) as specified in the SEYLE protocol, to be able to provide professional help and back-up to adolescents in need.

## Discussion

By asking the field coordinators open-ended questions about their experiences we have been able to gain a deeper understanding of the implementation of the Awareness program, complete with its’ difficulties and real-life situations. Research shows that a self-administered instrument of open-ended questions is practical and useful tool for evaluation [25].

The major strength of the Awareness program as proclaimed by the coordinators is its’ subtlety in content and execution. When addressing sensitive issues such as mental health, risky life-styles and suicide, it is important not only to be cautious and sensitive to cultural differences, but also to personal histories. Awareness programs for adolescents that are both effective and culturally adaptable need to be carefully developed, considering attitudes towards suicide and mental healthcare in general. Moreover, suicidal behaviors vary across countries, by gender and across the lifespan [26-28], with many other factors influencing these behaviors, such as a variety of cultural expressions, stigma, access to lethal means of suicide, lack of a medical/mental healthcare infrastructure; all of them usually linked. Risk and protective factors at the individual, as well as the larger societal level, need to be taken into consideration. A preventive effort specifically tailored for adolescents needs to be thorough in its approach; yet open to flexibility, allowing the youth to express themselves freely in a safe environment. Since it is very difficult to identify which adolescents are most at risk for suicide, increasing the general mental health awareness whilst encouraging youth to self-recognize the need for help, as well as to help peers in need, may lead to fewer suicides.

The SEYLE Awareness program helps the adolescents to develop a large set of skills and knowledge about mental-health: functional knowledge (knowing about Mental Health), procedural knowledge (knowing how/having skills) and conditional knowledge (knowing the circumstances in which to use the skills). This know-how is expected to lead to a heightened responsiveness to individuals with psychiatric, behavioral and/or emotional problems, or suicidal individuals, whilst diminishing the general stigma surrounding mental health. Accounts from the field demonstrate that the pupils not only learned new information by participating in the Awareness program, but the majority of them greatly enjoyed the experience.

Schools provide a well-structured environment that allows large international interventions, such as the SEYLE Awareness program, to run according to a *priori* defined rules that can be compared across different countries in spite of the many potential, imagined and real cultural differences. Though the school environment provides us with the best setting for programs aimed at adolescents, it is by no means an easy system to navigate and one of the more difficult aspects of the Awareness program was, in fact, the enormous organisational effort required from of the coordinators and their teams working with many different schools and teachers, during a short time period, especially to achieve adequate time in the school curriculum. Of course, the conditions of a study are in many ways much different than those encountered in real life, and we recommend that future Awareness programs take into account the problems encountered in the SEYLE field and the suggestions given here. The structured nature of the current program is inherent to a research study, but in a real-life setting, the time frame for each of the topics raised could be more flexible according to the specific class context and issues raised during the session. The incorporation of video materials and other types of learning methods may also be effective, but needs to be evaluated.

Suppleness in organisation and structure and listening to the thoughts and wishes of the participating adolescents is key to a successful program. The Awareness program is highly contextual and the feedback from the coordinators shows that the local context significantly influences the outcome of the program; every classroom is different, and consequently flexibility is central to a successful implementation. In the case of the SEYLE randomized controlled trial, it was necessary to execute the program in a structured manner to allow for effective comparison across sites through standardised methods. Much of the criticism from the Awareness coordinators dealt specifically with the more rigid aspects of the program; specifically the time frame but also the posters that were deemed too conspicuous in relation to other more adaptable aspects of the program.

In summary, the following guidelines can be helpful for people working with youth mental health awareness:

• Prepare a well-structured program with clearly defined aims, but allow flexibility and an individual approach.

• More time should be allocated to the variety of topics raised and to role-play and discussions with at least an additional five hours added to the program, resulting in a ten-hour program.

• Facilitators need to have a proper professional background and training, but also need to have specific personality traits (e.g. openness, ability to listen and make quick decisions) to create a safe environment.

• Topics should be addressed in a way that gives an opportunity to develop problem-solving skills and empathic attitude whilst creating an enjoyable and inspiring experience. Difficult topics should not be avoided, rather need to be addressed with care and close involvement of the participants.

• The key messages need to be disseminated through different materials and tools of interaction.

• Cooperation, understanding and support from stakeholders are crucial for success; the school system is the most effective system to use. Therefore, logistical issues (schedules, size of group, etc.) need to be tailored according to the needs and available resources.

• Holding an information event prior to the Awareness program to encourage teachers and parents to allow the children to participate by providing them with the opportunity to gain a better understanding of the aims and benefits of the program is beneficial. This informal meeting gives the parents an opportunity to meet the awareness coordinator and helps to demystify the program and make it more tangible for parents.

• Evaluation of the program should be done with pre-post assessments and also with process-evaluation.

### Limitations of this paper

The above-mentioned suggestions for successful awareness programs only take into account the issues raised by the set of questions the SEYLE Awareness coordinators were asked. For a more profound understanding of the program and it’s successes and failures, similar questions need to be asked to the participating adolescents as well as to teachers and other school personnel.

Only questions with open-ended answers were used in this evaluation. On the one hand, this enabled us to gather a variety of unexpected answers, important for exploring the field experiences. On the other hand, this approach limited the measurable comparisons of the responses to the same items, which is possible when using visual analogue scales. Importantly, this limitation was countered by using a systematic and rigorous approach in the content analysis of the material and summarising results in a meaningful way.

## Conclusions

The SEYLE Awareness program was developed with a large heterogeneous group of adolescents in mind. The main goals, to increase general mental health awareness whilst encouraging youth to self-recognize the need for help, were, of course, very ambitious. In such large-scale efforts, it is difficult to ensure that the needs of all participants are addressed and that all topics raised are adequately explained and actually understood. However, reports from the SEYLE sites in 11 European countries show that the adolescents not only learned about mental health by participating in the Awareness program, but that it was also an enjoyable and inspiring experience. The role-play sessions and ensuing discussions were a welcome diversion from ordinary classes and as such an excellent tool for communicating knowledge and diminishing mental health related stigma. Different from many other school endeavours, the program engendered understanding between pupils, encouraged peer support and allowed the pupils to get to know each other better, hopefully them to understand that they are not alone with their problems.

The school-environment is the best system we have to perform primary prevention programs designed to improve mental health and give information about unhealthy life-styles among youth, whilst at the same time raising the general awareness-level about mental health and mental problems. However, the help and support of schools, local politicians and other stakeholders, along with teachers, parents and adolescents, is needed for efficacious implementation of forthcoming awareness programs. Finally, the healthy functioning and understanding of mental health related issues for children and adolescents have profound consequences for society, both presently and in the future. Therefore, our expectations for the future are that comparable mental health and suicide preventive awareness programs will be included in the curriculums of schools across Europe.

## Competing interests

The authors declare that they have no competing interests.

## Authors' contributions

CW ideated and developed the content of the Awareness intervention program, coordinated the implementation, trained the personnel, analyzed the data and jointly drafted the manuscript. CH ideated and developed the content of the Awareness intervention program, coordinated the implementation, trained the personnel and contributed to the manuscript for relevant intellectual content. DW is the principal investigator of SEYLE, ideated and developed the content of the Awareness intervention program, coordinated the implementation and contributed to the manuscript for relevant intellectual content. VC coordinated the implementation of SEYLE, trained the personnel and contributed to the manuscript for relevant intellectual content. SAH, LF, GF, NG, DCH, MI, JMC, JZ led the Awareness intervention program at the respective study sites, collected the information presented in this manuscript and contributed to the manuscript for relevant intellectual content. DF, KK, PV, AT collected the information presented in this manuscript and contributed to the manuscript for relevant intellectual content. AA, JB, JB, DC, CH, JPK, HK, AV, MS are site leaders of SEYLE in the respective countries. They coordinated and provided support to the implementation of the Awareness intervention program in the respective study sites and contributed to the manuscript for relevant intellectual content. VP was site-coordinator and oversaw the implementation of the Awareness intervention program in Slovenia, analyzed the data and jointly drafted the manuscript with CW. All authors read and approved the final manuscript.

## Pre-publication history

The pre-publication history for this paper can be accessed here:

http://www.biomedcentral.com/1471-2458/12/776/prepub
